# Clinical study of endoscopic treatment of a sellar pituitary adenomas with sellar diaphragm defect

**DOI:** 10.1186/s12883-020-01690-8

**Published:** 2020-04-11

**Authors:** Zhuoru Jin, Xinyu Wu, Yibao Wang

**Affiliations:** The First Affiliated Hospital of China Medical University, No.155, North Nanjing Street, Heping District, Shenyang, 110001 Liaoning People’s Republic of China

**Keywords:** Endoscopic endonasal approach, Pituitary tumor, Saddle diaphragm, Suprasellar region

## Abstract

**Background:**

Invasive growth of pituitary macroadenomas to the suprasellar region occurs commonly. Pituitary adenomas show varying growth patterns when the sellar diaphragm is absent, and they are often confused with other common tumors in the sellar region. This article explores the clinical features of suprasellar pituitary adenomas with defects of the sellar diaphragm (SPADSD) and evaluates the efficacy of the endoscopic endonasal approach (EEA) for treatment of such tumors.

**Methods:**

We performed a detailed examination of records from 19 patients collected prior to surgery. After relevant diseases were excluded, the tumor properties were evaluated according to imaging characteristics. Diagnoses were verified using EEA surgery. The concept of SPADSD was put forward. Postoperative recovery was followed to determine whether EEA is suitable for the treatment of such tumors.

**Results:**

In the 19 patients with SPADSD, we found that the tumors were less stressed on the pituitary, and tumors in the suprasellar region often had irregular shapes. During surgery, we took extended supra-saddle approaches and confirmed that unrestricted growth of the tumor was caused by defects in the diaphragm of the sella turcica to the suprasellar region. Recovery was good after surgery, confirming the efficacy of EEA for treatment of these tumors.

**Conclusion:**

SPADSD has different clinical features from those of other pituitary tumors and requires careful screening prior to surgery. Endoscopic surgery is the preferred procedure for this type of tumor.

## Background

Pituitary adenoma is the most common tumor in the sellar region. The incidence of intracranial tumors ranks third, accounting for 10 to 15%, among all intracranial tumors [1, 2]. Studies have shown that the prevalence of pituitary tumors is about 5/100000 [3, 4]. Pituitary adenomas smaller than 10 mm are generally called pituitary microadenomas, those that are 10–30 mm tumors are called pituitary macroadenomas, and tumors larger than 30 mm are called pituitary giant adenomas [1].

Currently, there are two ways to grade pituitary adenomas using preoperative MRI. one is the Knosp classification [5]. This grading criterion is based on the position of pituitary adenomas relative to the internal carotid artery. The other is the modified Hardy classification (hereafter referred to as “Hardy classification”), which is used when pituitary adenomas break through the suprasellar cistern. The Hardy grading standard describes the degree of suprasellar extension of the tumor: Grade 0, the tumor remains in the saddle; Grade A, the tumor expands to the suprasellar cistern; Grade B, recessus opticus block; Grade C, the third ventricle is significantly displaced; Grades D are defined as the extent of the tumor above 20 mm on the sphenoid bone [6]. Large pituitary adenomas and pituitary giant adenomas usually protrude from the saddle area or even to the front of the skull base or the third ventricle [7]. According to the Hardy classification, tumors above the suprasellar cistern are referred to as suprasellar pituitary adenomas. However, in actual clinical work, a very small number of patients with saddle pituitary tumors have a congenital diaphragm sella turcica defects or serious defects in the diaphragm sella turcica. These conditions make the tumors originating in the sella have no pressure effect, and they grow irregularly to the suprasellar region. The tumor can invade the optic chiasma forward and squeeze the third ventricle floor upward. We call this a suprasellar pituitary adenoma with defect of sellar diaphragm (SPADSD). Non-functional pituitary tumors are diagnosed when the volume grows to a large extent and causes symptoms such as decreased vision [8, 9].

Transsphenoidal surgery is a commonly used to treat pituitary adenomas [10–13]. In recent years, there has been popular application of neuroendoscopy. This technique has the advantages of clear visual field, less blind area, less trauma, flexible observation angle, high total tumor resection rate, rapid recovery after surgery, and others. The endoscopic endonasal approach (EEA) has become the main surgical method for resection of pituitary adenomas [14]. Its excellent postoperative results have shown that this surgical procedure is effective not only for saddle adenomas but also for the giant pituitary adenomas invading the suprasellar region. At present, EEA is the treatment of choice for pituitary tumors that invade the suprasellar region [15].

Nevertheless, it is unclear whether EEA is safe and effective for treatment of SPADSD. Therefore, this article describes the clinical features of 19 patients with SPADSD treated with endoscopic surgery. We elucidated the imaging and anatomical features of these tumors to distinguish them from other tumors, summarizing the problems that should be addressed during surgery, and evaluating the efficacy of EEA.

## Methods

### Patient selection

From 2009 to 2019, at the First Affiliated Hospital of China Medical University, we performed a total of 1956 surgeries on patients with pituitary adenomas using EEA. We selected 19 patients (8 males and 11 females, age range: 26–81 years) whose tumors grew into the suprasellar region because of defects of the sellar diaphragm. According to the pathological report, other types of tumors were excluded, and the selected resected lesions were confirmed to be pituitary adenomas. All patients underwent enhanced MRI and skull base 3D-CT scanning at the First Affiliated Hospital of China Medical University.

#### Brain MRI

MRI data was acquired using a 3.0 T whole body MRI scanner (General Electric Medical System, GE Signa HDxt). Subjects were required to remain in a fixed position in the scanner. We placed a foam pad on either side of the head to reduce movement and used cotton ear plugs to reduce noise. Three-dimensional T1-weighted images obtained from the brain volume imaging (Bravo) sequence had the following parameters: echo time = 3.325 ms, repetition time = 8.0 ms, inversion time = 430 ms, layer thickness = 1 mm, flip angle = 15, excitation times = 1, voxel size = 0.5 · 0.5 · 1.0 mm^3^, field of view = 240 · 240 mm^2^, and acquisition time was 200 s.

#### Operative procedure keys

In the process of opening the sellar floor, the sellar bony markers such as the optic nerve eminence, the internal carotid artery eminence, the opto-carotid recess (OCR) and the clival recess (CR) were identified. We ground the sellar floor along the mediastinum of the sphenoid sinus on both sides, the lower part was ground to the CR, and the upper part was ground to the tuberculum. We used micro-scissors to cut the dura mater radially, revealing the lower end of the field of vision, and releasing the arachnoid exposed to the optic chiasm, revealing the whole tumor. A conventional 0° endoscope was used to sequentially remove the intra-saddle tumor, the parasagittal tumor, and finally the suprasellar tumor. The normal pituitary was identified and was protected according to the thin-enhanced image of the saddle area shown by the preoperative enhanced MRI. For the medial aspect of the cavernous sinus or the part of the internal carotid artery, transcranial doppler (TCD) was used to identify blood vessels before resection, and the part that broke through the saddle diaphragm to invade the skull base or invaded the third ventricle was removed with a surgical instrument at a certain bending angle, combined with 30° angle endoscopy to carefully determine whether there was any residual tumor. Intraoperative hemorrhage is one of the main complications of endoscopic surgery. Hemostasis was achieved by raising the patient’s head, filling the fibrils to compress the bleeding point, injecting fluid gelatin or bio-protein glue. Meninges, autologous fat, fascia lata, free and vascular mucosal flaps, and finally a Foley balloon were used in multi-level repair of the saddle floor for skull base reconstruction.

## Results

**Fig. 1:**
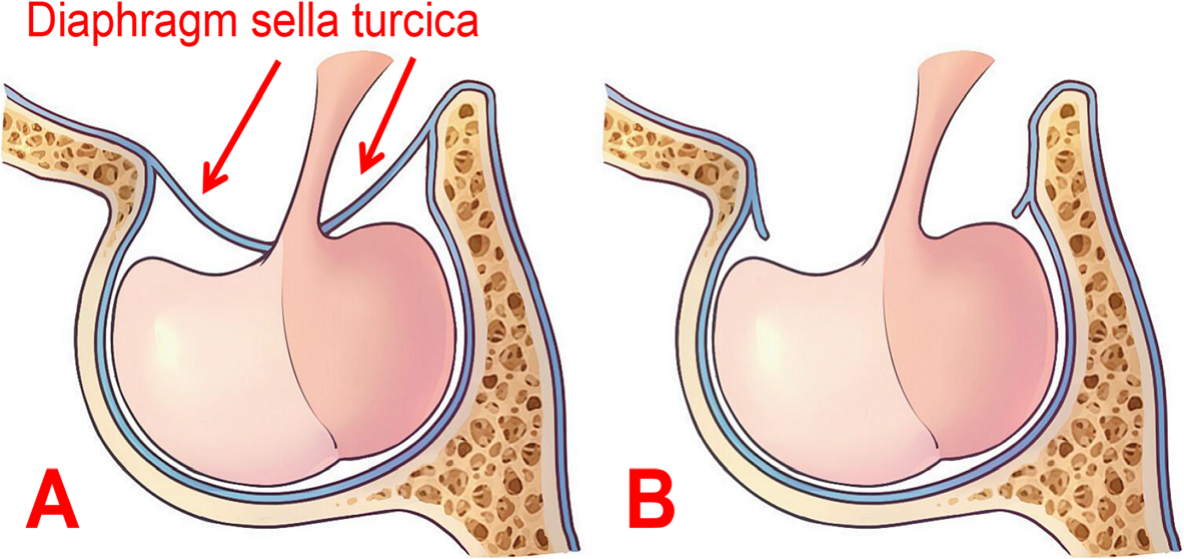
**a** Schematic diagram of the shape of a normal pituitary crypt. The red arrow identifies an intact saddle, and there is no defect. **b** Schematic diagram of a pituitary crypt caused by a congenital defect of the saddle diaphragm, with the saddle diaphragm is already missing

**Fig. 2:**
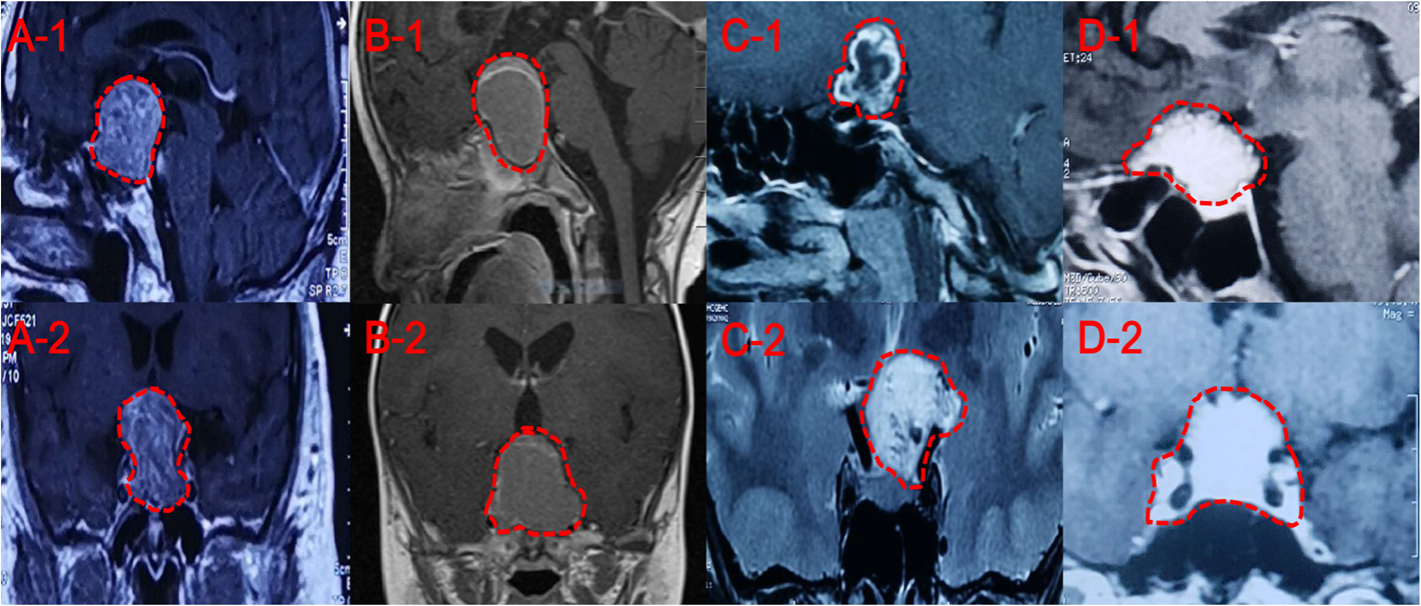
Four common tumors in the sellar region. These tumors often mimic SPADSD. (**a**-1**, a**-2) MRI of a patient with a pituitary tumor growing in the suprasellar region. The mass shows uneven enhancement, the optic chiasm is elevated, and the pituitary is compressed at the left posterior of the tumor. The tumor appears “hoisted”. (**b**-1, **b**-2), MRI of a patient with a cystic solid craniopharyngioma in the sellar region and suprasellar region. There is visible enhancement at the edge of the lesion and no enhancement in the sac. The pituitary stalk cannot be observed and the optic chiasm is elevated. (**c**-1, **c**-2) MRI a patient with a hair cell astrocytoma in the suprasellar region. It shows enhancement of the visible lesion edge and patchy enhancement in the lesion. The pituitary stalk and optic chiasm are under pressure, and there is no abnormality in pituitary morphology. (**d**-1, **d**-2) MRI of a patient with a saddle nodular meningioma. The tumor showed a uniform enhancement, revealing the meningeal tail sign. The optic chiasm, pituitary morphology, size and signal all show no abnormalities. The area enclosed by the red dotted line in the eight images is the area where the tumors grow

**Fig. 3:**
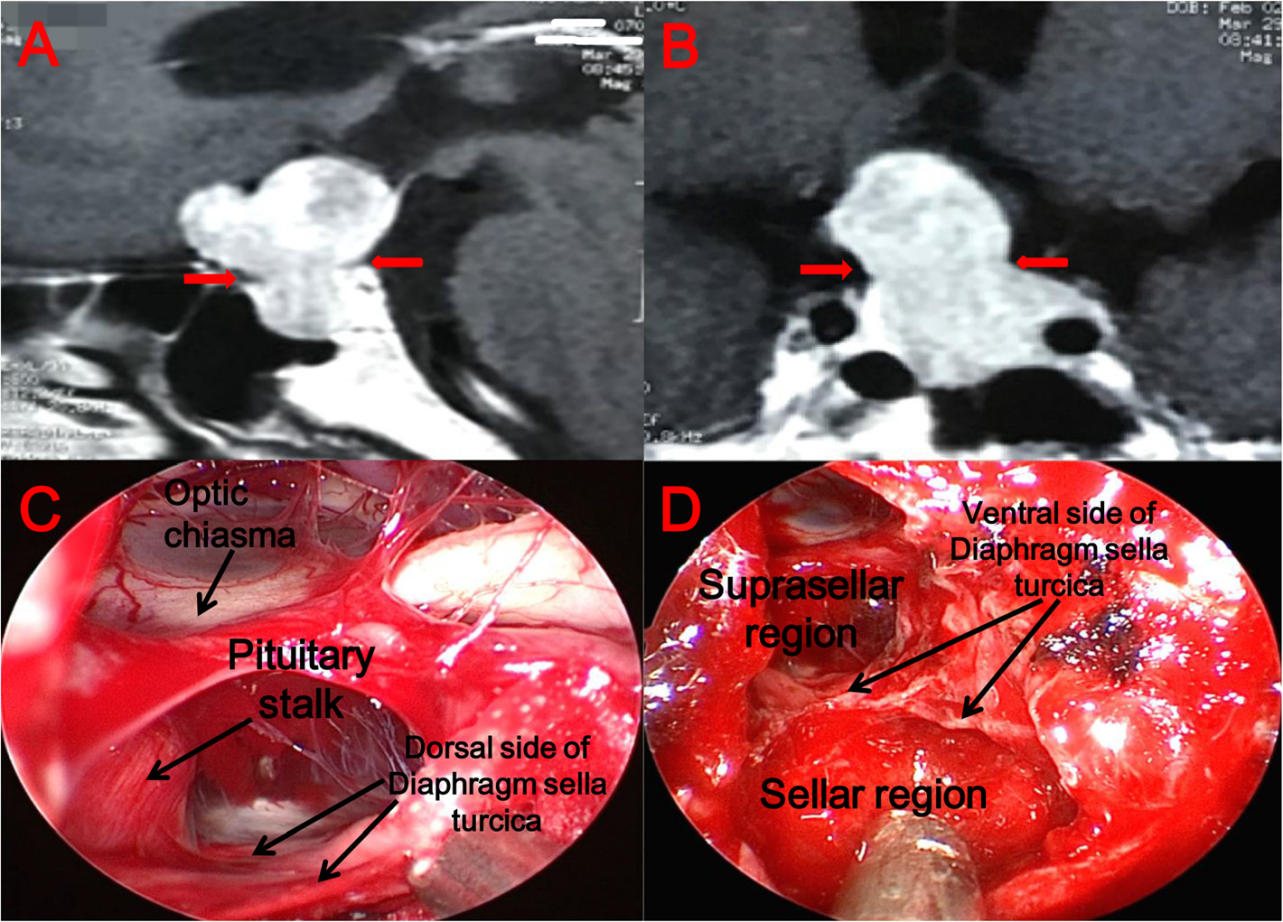
A patient with non-functional pituitary macroadenoma. (**a, b**) Sagittal and coronal views on MRI. The area pointed to by the red haircut is the place where the tumor is bound by the Diaphragm sella turcica. (**c, d**) These are the images during the operation, and we can see clearly the existence of Sellar diaphragm

All 19 patients underwent surgery using EEA. During the procedure, we confirmed that these patients suffered from congenital loss of the diaphragm sella turcica (Fig. 1a, b), causing the tumor to invade from the saddle to the suprasellar region. Because growth characteristics and MRI signal feedback are similar to those of many common tumors in the saddle area, it is easy misdiagnose the tumor preoperatively (Fig. 2). Compared with the integrity of the sellar diaphragm, the defect of the diaphragm sella turcica allows growth of the tumor to be unrestricted; therefore, it is more likely to invade tissues of the suprasellar region such as the optic nerve. However, in clinical practice, we found that some non-functional pituitary tumors are invasive, they can break through the diaphragm sella turcica and grow into the suprasellar region. Because of the characteristics of this tumor, the growth pattern of this kind of pituitary tumor is easily confused with SPADSD (Fig. 3). As we can see in Fig. 3, because the tumor always originates in the Sellar region, and the diaphragm sella turcica above the tumor hinders the growth of the tumor to the suprasellar region, it will oppress the hypophysis and so on. It is difficult to find the normal shape of the pituitary gland in the MR image, even the flattened pituitary gland can not be found (Fig. 3a, b). When the diaphragm sella turcica is destroyed, it will provide a small channel for non-functional pituitary tumors to grow into the suprasellar region. But the diaphragm sella turcica is almost not completely destroyed, so we will find a waist-shaped shape of the tumor on the level of the diaphragm sella turcica on MR images (Fig. 3a, b). Through the images during the operation, we did find the existence of the diaphragm sella turcica and confirmed our conjecture (Fig. 3c, d).

After the operation, 6 patients developed symptoms of cerebrospinal fluid leak (CSF). Four of patients recovered on their own within 3 days after the occurrence of CSF, the other two patients had no improvement in the symptoms of CSF and developed symptoms of infection such as fever and chills, so we performed surgery on these two patients to repair CSF. All 19 patients were discharged from hospital and there were no deaths due to factors such as intracranial infection (Table 1).

**Table 1:** Case summary

Age (years)	Number of cases	Gender		Abnormal hormones					Degree of resection of the tumor		Cerebrospinal fluid leak	
		Male	Female	ACTH	GH/IGF-1	PRL	TSH	Other	Entire	Partial	No repairing	Repairing
20~29	1	0	1	0	0	0	1	0	1	0	0	0
30~39	2	1	1	0	0	1	0	1	2	0	1	0
40~49	4	1	3	1	0	1	0	2	4	0	0	1
50~59	5	3	2	0	2	1	1	1	4	1	1	0
60~69	3	1	2	0	1	0	0	2	2	1	0	1
70+	4	1	3	0	0	2	0	2	4	0	2	0
Total	19	7	12	1	3	5	2	8	17	2	4	2

Mean age = 55.1 ± 14.7 Median age = 55

**Fig. 4:**
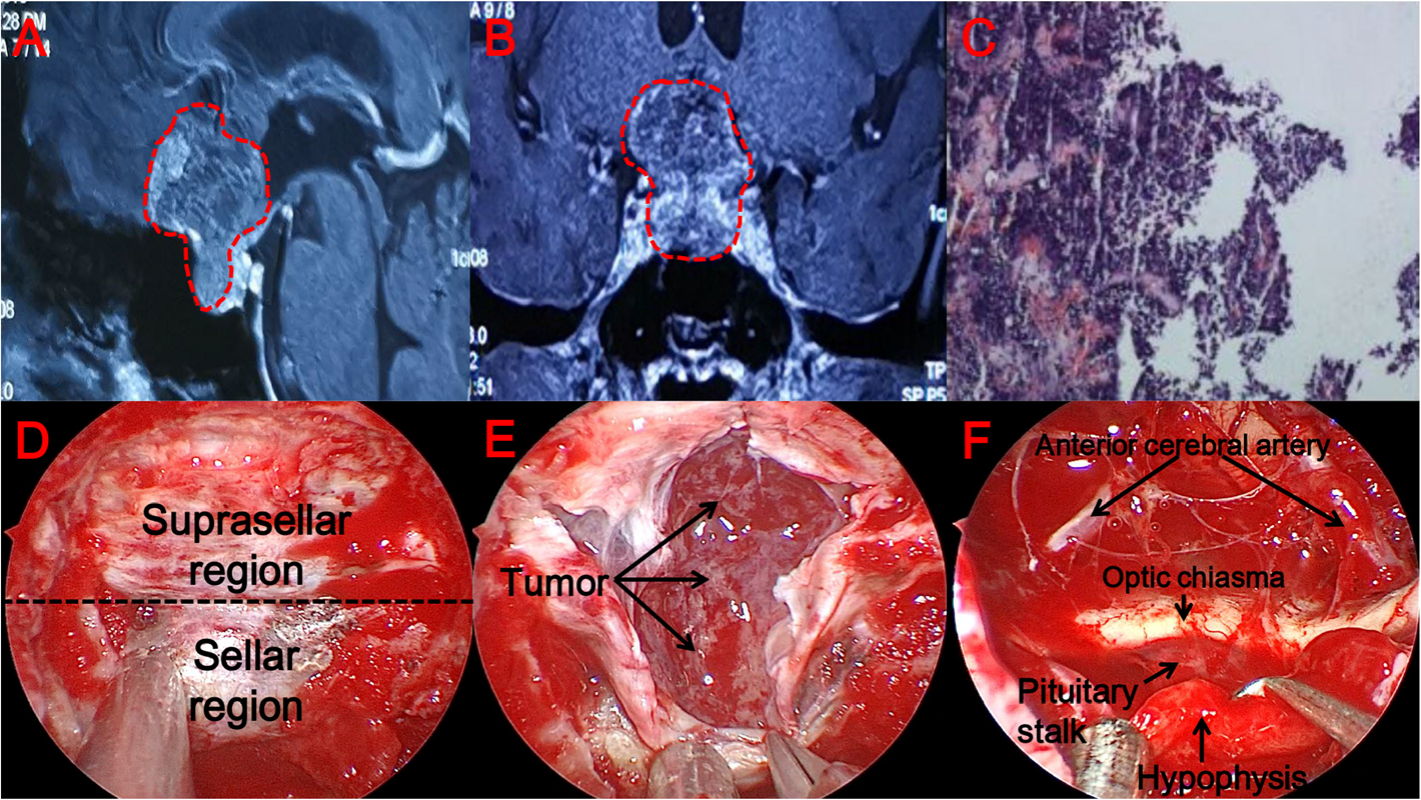
Patient 1 is a woman. (**a, b**) Sagittal and coronal views on MRI. The solid mass is in the saddle and suprasellar area, showing uneven enhancement. The optic chiasm is elevated and the pituitary is compressed in the right and rear area of the mass. The area surrounded by the red dotted line is the area where the tumor is located. (**c**) Postoperative pathology, tumor cells diffuse in a patchy distribution, local distribution around the sinusoids, cell morphology more consistent with nuclear deep staining, cytoplasmic dichromatic and part of the cytoplasm is stained red. (**d**) After removal of the saddle floor bone, we can see the boundary between the saddle and suprasellar area. (**e**) A soft tumor is observed under the microscope during surgery. (**f**) An image of the surgical area after removal of the tumor. The surrounding important adjacent structure is not damaged

Patient 1 is a woman (Fig. 4a, b). One month before the operation, the patient developed bilateral blurred vision and a right-sided vision defect; nevertheless, she did not undergo treatment. One week before the operation, the patient suddenly developed a sharp headache with blindness in the right eye. After two hours, the patient’s symptoms improved spontaneously. Before the operation, we performed brain MRI, as well as eye, pituitary and thyroid function hormone tests. We first ruled out diseases of the eye. Because pituitary-related hormones were within normal ranges, the tumor was thought to be a non-functional pituitary adenoma. After completing the relevant examinations, we performed surgery. During the operation, we used the grinding stone to remove saddle bone, and identified the dura mater (Fig. 4d). We used TCD to determine the position of blood vessels and the cut the dura mater sharply, revealing the tumor. The texture of the tumor was soft (Fig. 4e). The pituitary morphology remaind intact during the operation. After the tumor was completely removed, no damage was caused to the surrounding optic nerve and anterior cerebral artery (Fig. 4f). We used a middle turbinate pedicled mucosal flap, artificial dura mater and fibrin-thrombin glue to repair the area. After surgery, the patient’s visual acuity improved significantly, and the visual field defect on the right side resolved. On the fourth day after surgery, the patient developed symptoms of cerebrospinal fluid (CSF) leak; however, there were no symptoms related to intracranial infection. After repair of the CSF leak, the patient had no more symptoms referable to the CSF symptoms. There was no hypophysis dysfunction after the operation. The patient was discharged from the hospital on the 10th day after surgery. Pathology after surgery confirmed that the tumor was a pituitary adenoma. Immunohistochemical index of pathology: CK(+), ACTH(±), HGH(−), PRL(−), Synaptophysin(+), P53(−), Ki-67(5%+) (Fig. 4c).

**Fig. 5:**
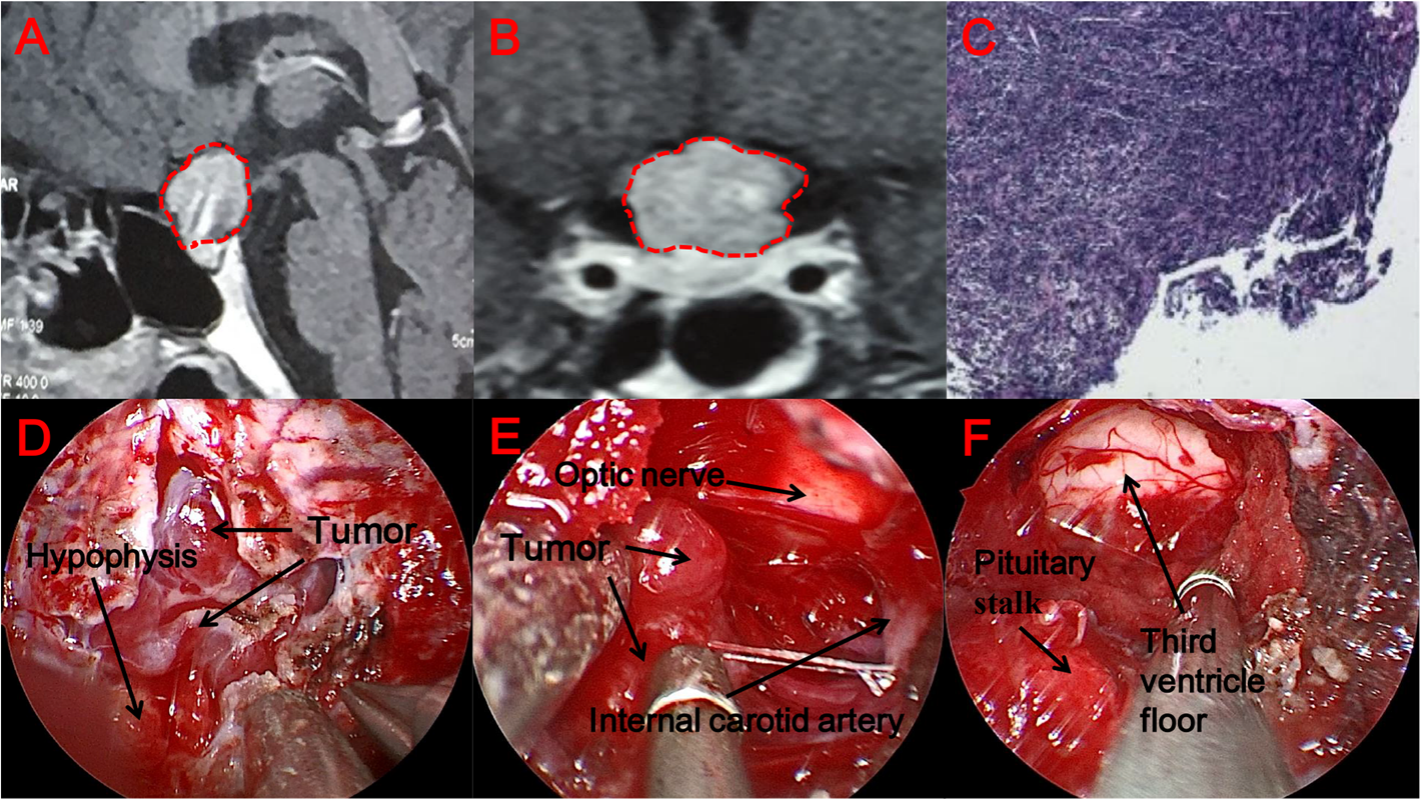
Patient 2 is a man. (**a, b**) Coronal and sagittal views on MRI. The solid mass is in the saddle and suprasellar area, showing uneven enhancement. The optic chiasm is elevated and the pituitary is compressed in the right area of the mass. The area surrounded by the red dotted line is the area where the tumor is located. (**c**) Postoperative pathology: tumor cells diffusely in a patchy distribution, local distribution around the sinusoids, cell morphology more consistent with nuclear deep staining, cytoplasmic dichromatic and part of the cytoplasm staining red. (**d**) After removing the bone of the saddle and sharply cutting the dura mater, we found a hard tumor and identified the pituitary. (**e**) During the process of excising the tumor, the important anatomical structure of the adjacent tumor is clearly visible. (**f**) An image of the surgical area after removal of the tumor. The surrounding important adjacent structure is not damaged

Patient 2 is a man (Fig. 5a, b). The patient had a first operation in another hospital two years prior because of dizziness and palpitations. The surgery consisted of removal of 10% of the tumor. The patient had decreased visual acuity one month prior to admission to our hospital. After MRI, it was determined that the tumor had recurred. We tested hormones related to pituitary and thyroid function before surgery, revealing FT4: 27.68 pmol/L, FT3: 14.84 pmol/L, TSH: 5.24 mIU/L; all of which were higher than the normal values of 8.63 pmol/L, 9.14 pmol/L and 0.29 mIU/L, respectively. After we excluded diseases related to the eyes and heart, we performed surgery. We ground off the saddle bone, and identified the dura mater directly. After we used TCD to determine the position of the blood vessels, we used microsurgical scissors to sharply cut the dura mater and identify the tumor (Fig. 5). The tumor was tough and its blood supply was abundant. We bluntly separated the tumor from the surrounding important anatomical structures, and found that the pituitary morphology remained intact during the operation. After tumor resection, the optic nerve, anterior cerebral artery and the third ventricle were clearly seen to be free of damage (Fig. 5e, f). We used a middle turbinate pedicled mucous flap, artificial dura mater, and fibrin-thrombin glue to repair the surgical area. Postoperative self-reported visual acuity improved after surgery. On the third postoperative day, we performed pituitary-related hormone and thyroid function tests, notable for FT4: 18.73 pmol/L, FT3: 3.30 pmol/L, and TSH: 0.71 mIU/L. These three indicators were all within the normal ranges. Postoperatively the patient had no symptoms of CSF leak, intracranial infection, or hypophyseal dysfunction. The patient was discharged from the hospital on the 7th postoperative day. The pathology after surgery confirmed that the tumor was a pituitary adenoma. Immunohistochemical index of pathology: CK(+), ACTH(−), HGH(+), PRL(−), Synaptophysin(+), P53(−), Ki-67(10%+), S-100(−) (Fig. 5c).

**Fig. 6:**
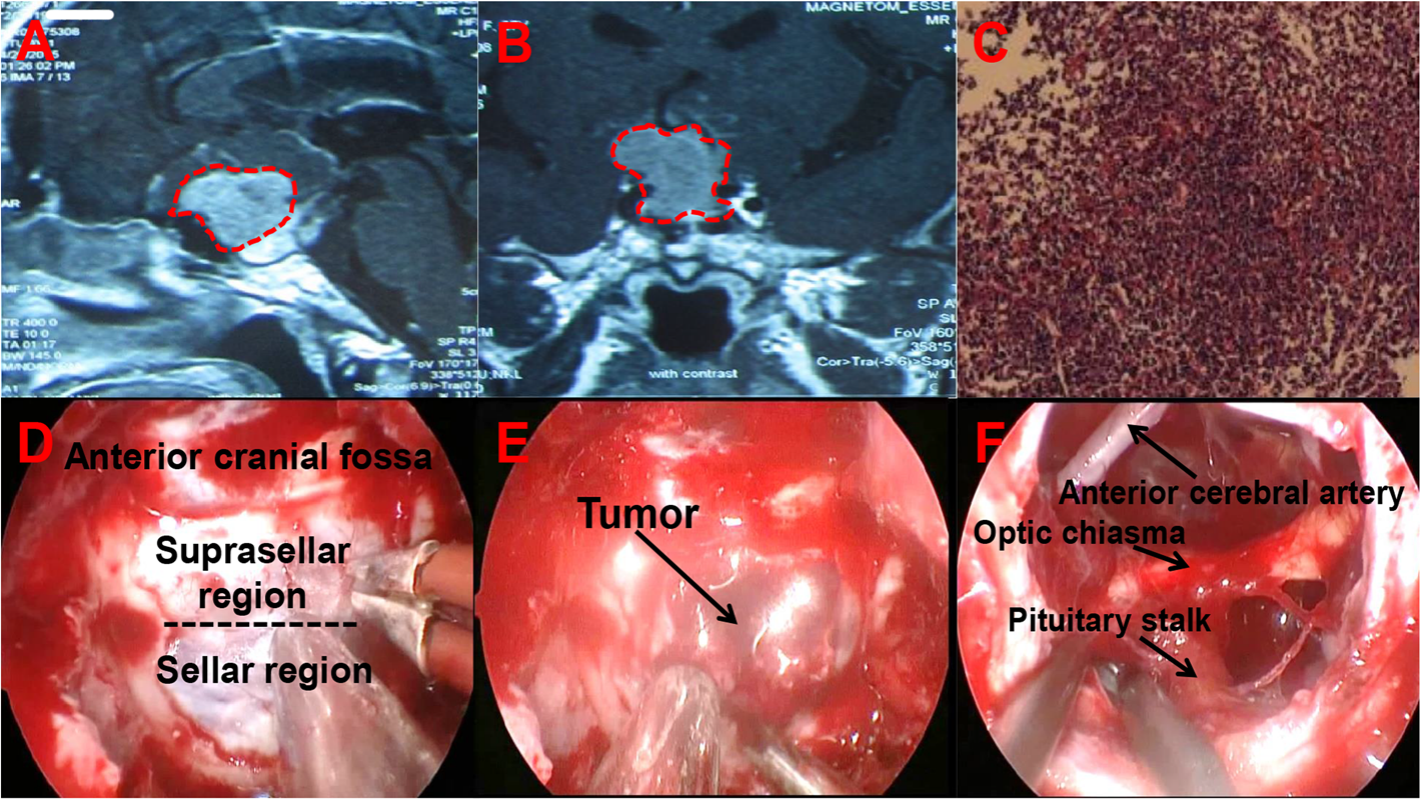
Patient 3 is a woman. (**a, b**) Sagittal and coronal views on MRI. The solid mass is in the saddle and suprasellar area, and the suprasellar apart appears lobulated. The tumor shows uneven enhancement. The optic chiasm is elevated and the pituitary is compressed by the mass. The area surrounded by the red dotted line is the area where the tumor is located. (**c**) Postoperative pathology, tumor cells diffuse in a patchy distribution, local distribution around the sinusoids, cell morphology more consistent with nuclear deep staining, cytoplasmic dichromatic and part of the cytoplasm staining red. (**d**) After removal of the saddle floor bone, we can see the boundary between the saddle and suprasellar area. (**e**) A soft tumor is observed under the microscope during surgery. (**f**) An image of the surgical area after removal of the tumor. The surrounding important adjacent structures are not damaged

Patient 3 is a woman (Fig. 6a, b). The patient began to experience vision loss one year prior. In the two weeks prior to admission, her visual acuity degraded severely. Only some light could be seen before surgery, and no objects in front of the eyes could be seen. After exclusion of eye-related diseases, the patient underwent MRI, revealing high likelihood of a pituitary tumor. Before the operation, we performed examinations of pituitary and thyroid function-related hormones; all hormones were within the normal ranges. During surgery, we used a grinding drill to open the saddle bottom. After the bone was removed, revealing the dura mater (Fig. 6a, b). After using TCD to determine the position of the blood vessels, the dura mater was sharply cut, revealing that the tumor texture was soft and the blood supply was not abundant (Fig. 6e). The tumor was completely removed under microscopy. The optic nerve and the anterior cerebral artery were shown to be intact and the pituitary morphology remained intact (Fig. 6f). We used a middle turbinate pedicled mucosal flap, artificial dura mater, and fibrin-thrombin glue to repair the area. On the second day after surgery, the patient could see objects in front of his eyes. There were no CSF leak, intracranial infection, or hypophyseal dysfunction after surgery. The patient was discharged from hospital on the 7th day. Pathologically confirmed after surgery that the tumor was a pituitary adenoma. Immunohistochemical index of pathology: CK(+), ACTH(−), HGH(−), PRL(±), Synaptophysin(+), P53(−), Ki-67(3%+) (Fig. 6c).

We followed up 19 patients half a year after the operation. None of the 19 patients had recurrent CSF, only one patient had no obvious recovery of visual impairment, and only one patient had hypofunction of hypophysis.

## Discussion

Through our study, we found that it can be judged before operation according to the characteristics of SPADSD, and reduce the probability of wrong diagnosis. SPADSD often grows on the saddle because there is no physical restraint. These tumors do not compress the pituitary, but rather they grow toward the upper unrestricted area. On MRI, we often note that the shape of the pituitary is normal. Nevertheless, the shape of the SPADSD is usually irregular, showing a lobular shape. There are few violations of the pituitary during surgery, and there are fewer cases of hypofunction of the pituitary after surgery.

SPADSD is an invasive pituitary tumor that manifests in several invasive modes. It can break through upwards towards the saddle to invade the anterior skull base or the third ventricle, and can invade the cavernous sinus or can surround the internal carotid artery growing to the saddle sides. At present, there is no relevant research demonstrating which surgical method is more suitable for the resection of the SPADSD. We combined with the advantages of endoscopic techniques to conduct an in-depth discussion and review of the treatment of this tumor. The growth patterns and imaging signals of SPADSD are often very similar to those other intracranial tumors; therefore, the clinician often has difficulty with the preoperative diagnosis and the choice of surgical methods. Below, we compare the characteristics of other common tumors in the sellar region with those of SPADSD, in hopes of reducing the misdiagnosis, better understanding their common growth patterns, and developing better treatment schemes.

### Other types of pituitary adenomas in the suprasellar region

On preoperative MRI, pituitary adenomas often exhibit equal or low signal in T1, equal or high signal in T2, and uniform enhancement signal on enhancement. Normal pituitary tissue, because of the compression of the saddle tumor, shows a thin layer arc on enhanced imaging of the saddle region. Because of the absence of sellar diaphragm compression, SPADSD exhibits imaging and anatomical features that are different from those of other pituitary adenomas in the suprasellar region. Despite the similar signal shown by SPADSD, there is no typical “waist sign” or “snowman sign.” Their shape is irregular, and their position is located more on the saddle with fewer components in the sellar region. Therefore, the pituitary can assume a normal shape.

During the operation, attention should be paid to the following points: 1) The patient should be positioned gently leaning back; 2) When entering the nasal cavity, the middle turbinate should be removed, and a vascular mucosa flap should be prepared; 3) A bone window should be ground at least above the saddle nodule position, even reaching the sphenoidal platform; 4) After opening the dura mater, it is often found that there is no saddle diaphragm when separating tumor, and the tumor is closely adhered to the optic chiasm. Therefore, the tumor must be carefully separated from the arachnoid membrane along its membrane; 6) For the position of the third ventricle, the surgeon should attempt to create an in situ separation to reduce the risk of postoperative hypothalamic nuclear damage; 7) one should avoid electrocoagulation so as not to burn hypothalamic small perforating artery; 8) If the third ventricle floor is not properly preserved after tumor resection, the ventricular system should be opened to liberate cerebrospinal fluid circulation; 9) At 6 h after surgery, the bleeding in the surgical area should be monitored so as to screen for acute hydrocephalus; 10) For reconstruction of the skull base after the extended transnasal approach, it is necessary to use multiple layers of repair to effectively prevent postoperative CSF.

### Craniopharyngioma

SPADSD needs to be differentiated from craniopharyngioma. The latter originates from the pituitary stalk, and the lesion is often located in the suprasellar region. The normal pituitary can usually be observed on MRI. Tumors often appear as cystic and solid, and fewer solid tumors are often similar to pituitary adenomas. The composition of craniopharyngioma is complex; calcification is commonly seen on CT, and the signal is variable in MRI. The cystic part may have low, equal or high signal due to varying protein content, high signal or low signal in T2. The solid part has equal signal in T1, and slightly higher signal in T2. In the enhanced phase, the tumor parenchyma and the wall of the capsule show uneven enhancement. During the intraoperative resection of craniopharyngioma, it is necessary to determine the relationship between the tumor and the pituitary stalk. When the trans-infundibular craniopharyngioma is matched, the pituitary stalk should not be retained. At this time, the identification of pituitary adenomas is particularly important. The treatment of pituitary adenomas should not permanently damage the pituitary stalk. This is particularly important for differentiation of SPADSD from craniopharyngioma.

### Tuberculum sellae meningioma

Roughly 8% of meningiomas occur in the saddle, and most originate from saddle nodules. On MRI, tumors show equal signals in T1 and T2, with clear boundaries, and a substantially uniform enhancement phase. The tumor grows along the meninges and on both sides, with frequent appearance of the meningeal tail sign. The difficulty in the treatment of saddle nodule meningioma is that the tumor is rich in blood supply and is connected to the surrounding blood vessels. Behind the optic chiasm, the adhesion is extremely tight. During treatment, particular attention should be paid to the relationship of the tumor to the superior pituitary and anterior cerebral arteries. The treatment of the relationship between the optic chiasm and the tumor is also applicable to SPADSD. Lacking effective dura mater or even separation of the arachnoid membrane, SPADSD is in direct contact with the optic chiasm. Therefore, during surgery, a bone window should be opened to the saddle nodule first, and the anterior skull base dura mater should be cut open to expose the optic chiasm that will lay the foundation for the subsequent step of treating the tumor behind the chiasm.

### Advantages of endoscopic surgery

Compared with traditional microsurgery, endoscopic surgery enjoys better illumination and better visual effects, with expansion of the visual range because of the use of angle mirrors. The high-resolution image more accurately distinguishes the saddle and the arachnoid, visualizing the important structures around the internal carotid artery, the third ventricle, and the pituitary and tumor tissues, thereby protecting the pituitary. It has great advantages for treatment of suprasellar tumors. According to statistics, the endoscopic resection rate of suprasellar pituitary adenomas defined by Hardy classification is 96% [16]. Abergel et al. compared patients undergoing EEA surgery and traditional craniotomy. They concluded that EEA had less impact on psychological and emotional aspects than did traditional craniotomy and patients enjoyed a better quality of life [17, 18]. Nevertheless, the endoscopic transnasal approach is technically challenging for most neurosurgeons and has a longer learning period. EEA often increases the incidence of CSF. Nevertheless, with the development of endoscopic technology, EEA will gradually be widely used by clinicians [19].

Through the review of the relevant literature in the past 5 years, we prove that EEA has more advantages than traditional surgery in the treatment of pituitary tumors through the results of three related articles, they all performed a systematic review and meta-analysis performed a systematic review and meta-analysis. In 2014, Gao et al. performed 15 studies (*n* = 1014 patients) among 487 studies that involved endoscopic surgery and 527 studies that dealt with microscopic surgery [20]. In 2015, Xu et al. evaluated 1888 patients from 14 studies and get the result that compared with microscopic group [21]. In 2017, Li et al. assessed 2272 patients with pituitary adenoma included in twenty-three studies [22]. Through the relevant data and statistics, they have proved that the rate of gross tumor removal was higher in the endoscopic group than in the microscopic group and the post-operative hospital stay was significantly shorter for the endoscopic surgery group. But there was no significant difference between the two techniques in the incidence rates of meningitis, diabetes insipidus, cerebrospinal fluid leak, epistaxis, hypopituitarism and the length of the operation.

We reviewed more than 1900 patients who underwent pituitary adenoma surgery; however, only 1% had SPADSD. These are similar to other tumors in terms of appearance and surgical approaches. We think it important to put forward this concept. Because the bone window of the surgical approach is wider than that of other pituitary adenomas, and even need to be ground above the saddle nodule position, otherwise, the suprasellar tumor will not be removed effectively. If the Sellar diaphragm exists, part of the suprasellar tumor might fall into the Sellar after resection of the intrasellar tumor. Unfortunately, there is no such one. So it was different and more difficult at the beginning of the surgery. Since there is no Sellar diaphragmatic septum during the operation, special attention should be paid to the protection of the anterior communicating artery and optic nerve. It is a kind of tumor with histological features of pituitary adenomas and similar to endoscopic surgery for craniopharyngiomas. The nature of the tumor has a decisive influence on the choice of access to the procedure, the degree of resection during surgery, and the degree of retention of important structures during the procedure. Therefore, it is important to understand the characteristics of the tumor as much as possible before surgery, because this plays an important role in the treatment of the disease.

## Conclusion

SPADSD has different features from other tumors growing in the suprasellar region, including growth pattern, imaging features, surgical features, etc. We discussed SPADSD in detail, and compared its growth patterns and imaging characteristics with those of similar intracranial tumors. After summarizing the surgical procedures of 19 patients, we believe that EEA is the preferred surgical approach to removal of such tumors.

### Limitations

Because SPADSD is relatively rare, it is impossible to show all the characteristics with only 19 cases. We need more cases to validate our conclusions. CSF is a common problem after transsphenoidal surgery. The protection of the dura mater and the repair of the surgical area during surgery also need to be solved in the future.

## Data Availability

The datasets used and analysed during the current study are available from the corresponding author on reasonable request.

## Abbreviations

SPADSD: Suprasellar pituitary adenomas with defects of the sellar diaphragm
EEA: Endoscopic endonasal approach
MRI: Magnetic resonance imaging
OCR: Opto-carotid recess
CR: Clival recess
TCD: Transcranial doppler
CSF: Cerebrospinal leak
